# Enantioseparation by crystallization using magnetic substrates†
†Electronic supplementary information (ESI) available: The full data set of the crystallizations, the measurements of the magnetic properties of the surface, the details on the theoretical calculations and the general experimental methods. See DOI: 10.1039/c9sc00663j


**DOI:** 10.1039/c9sc00663j

**Published:** 2019-04-10

**Authors:** Francesco Tassinari, Jakob Steidel, Shahar Paltiel, Claudio Fontanesi, Meir Lahav, Yossi Paltiel, Ron Naaman

**Affiliations:** a Department of Chemical and Biological Physics , Weizmann Institute of Science , 234 Herzl Street , Rehovot 76100 , Israel . Email: ron.naaman@weizmann.ac.il; b Department of Engineering “Enzo Ferrari” , University of Modena and Reggio Emilia , Via Pietro Vivarelli 10 , Modena 41125 , Italy; c Department of Materials and Interfaces , Weizmann Institute of Science , 234 Herzl Street , Rehovot 76100 , Israel; d Department of Applied Physics , Center for Nano Science and Nanotechnology , Hebrew University of Jerusalem , Balfour Street , Jerusalem 91904 , Israel

## Abstract

Enantiospecific crystallization of the three amino acids asparagine (Asn), glutamic acid hydrochloride (Glu·HCl) and threonine (Thr), induced by ferromagnetic (FM) substrates, is reported. The FM substrates were prepared by evaporating nickel capped with a thin gold layer on standard silicon wafers. Magnets were positioned underneath the substrate with either their North (N) or South (S) poles pointing up. Asymmetric induction, controlled by the magnetic substrates, was demonstrated for the crystallization of the pure enantiomers and was then extended for the racemic mixtures of Asn and Glu·HCl. In the case of the solution of the pure enantiomers, the l enantiomer was crystallized preferentially at one pole of the magnet and the d enantiomer at the other. Consequently, the racemates of Asn and Glu·HCl undergo separation under the influence of the magnetic substrate. With Thr, however, despite the enantiospecific interactions of the pure enantiomers with the FM, no separation of the emerging crystals could be achieved with the racemates, although they crystallize as conglomerates, implying differences taking place in the crystallization step. The results reported here are not directly related to the magnetic field, but rather to the aligned spins within the ferromagnets. The findings provide a novel method for resolving enantiomers by crystallization and offer a new perspective for a possible role played by magnetic substrates regarding the origin of chirality in nature.

## Abstract 2

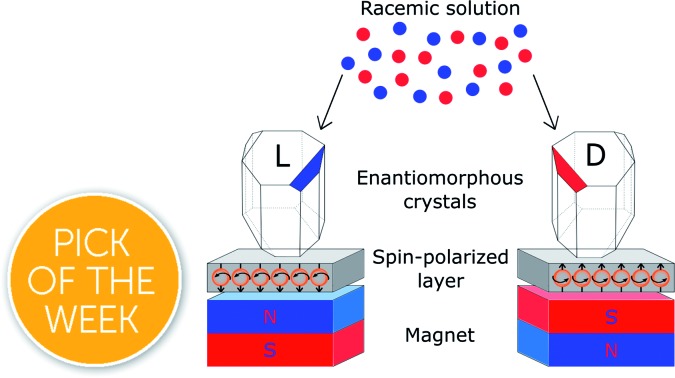
Enantiospecific crystallization of the three amino acids asparagine (Asn), glutamic acid hydrochloride (Glu·HCl) and threonine (Thr), induced by ferromagnetic (FM) substrates, is reported.

## Introduction

Biological systems are composed of molecules of a single chirality; hence, the origin of chirality in nature and the relationship between chirality and asymmetric forces, like magnetism, has challenged scientists in the past and is still the focus of much work at the present time.

Since the manual resolution of the enantiomorphous crystals of salts of tartaric acids by Pasteur,^1^,^2^ the question of how homochiral biopolymers emerged from a non-chiral prebiotic world attracts interest. Pasteur himself speculated that the magnetic field of Earth might have applied asymmetric induction in the emergence of the early chiral biopolymers.^3^ This hypothesis led him to attempt asymmetric reactions in strong magnetic fields. However, as demonstrated in early experiments by Faraday, magnetic fields *per se* are not chiral.^4^ Moreover, de Gennes has demonstrated that even the superposition of a magnetic field and an electric field, originally suggested by Curie,^5^ does not induce asymmetry in reactions at the final equilibrium state.^6^ The issue of “equilibrium *versus* non-equilibrium” was discussed in details by Barron.^3^ On the other hand, “absolute” asymmetric transitions can be performed when electromagnetic light is coupled with strong magnetic fields, as demonstrated in the experiments of Rikken *et al.*^7^–^10^

**Fig. 1:**
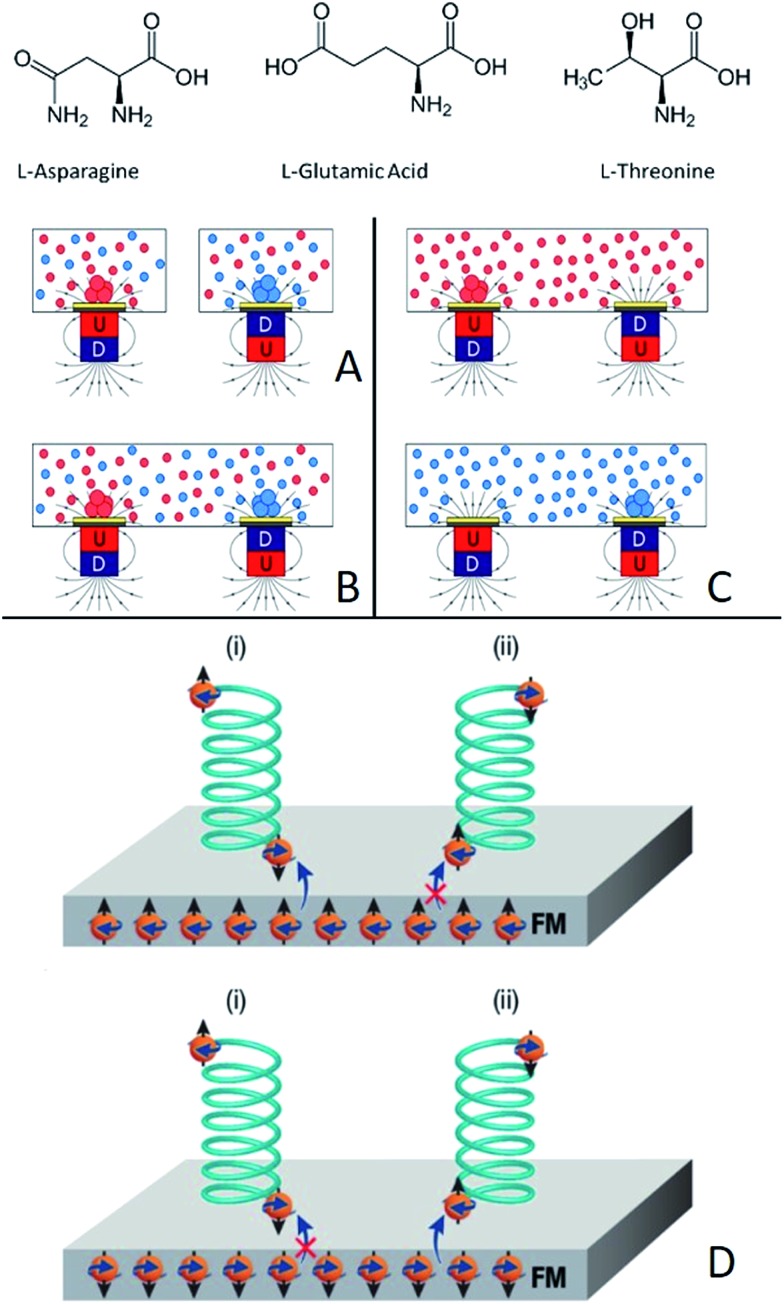
The three molecules (asparagine, glutamic acid, and threonine) described in the experimental setup. The experiments were conducted in three different configurations. (A) A magnet, either pointing with its North pole up (N) or down (S), is positioned underneath a ferromagnetic substrate (FM). The Ni(120 nm)/Au(10 nm) FM film was evaporated on silicon and points towards the racemic solution. Blue and red denote the S and N poles, respectively. (B) Two magnets, one oriented N and the other S, are placed underneath the Si/Ni/Au substrate in a racemic mixture. (C) Two magnets, one oriented N and the other S, are placed underneath the Si/Ni/Au substrate in an enantiopure solution. (D) The suggested mechanism for the enantioselective crystallization. When a chiral molecule approaches the ferromagnetic surface charge polarization occurs, accompanied by spin polarization. The interactions are stronger when the spins of the FM are aligned antiparallel to the spin of the chiral molecule. Therefore, the enantiomer with the stronger interaction has a longer residence time at the magnetic site and a greater chance to crystallize.

Recently we introduced a new concept related to interactions between magnets and chiral molecules. This concept is based on our observation that charge polarization in chiral molecules is accompanied by spin polarization^11^ and by the realization that the polarized spin in the chiral molecule interacts in an enantiospecific manner with ferromagnets (FM) that have their spin aligned perpendicular to their surface,^12^ as shown in Fig. 1D. It is important to realize that the interaction is not due to the magnetic field itself but, rather, to the interaction between electrons in the substrate and in the molecules *via* the electronic spin exchange interaction. This interaction is about several tens of kJ mol^–1^ at molecule-surface distance of 0.1–0.2 nm, as found by *ab initio* calculations.^11^ Here we present a method that allows induction of enantiopure crystallization (and separation) based on the interaction of chiral molecules with a magnetic substrate, magnetized perpendicular to its surface. By applying this concept, we can actually separate enantiomers spatially by applying magnets to enantiomorphous crystallization, where the enantioselectivity depends on the magnetic field direction.

## Results and discussion

We describe the magnetic substrate-induced enantiopure crystallization of three amino acids: asparagine (Asn), glutamic acid hydrochloride (Glu·HCl), and threonine (Thr). All three are known to crystallize as conglomerates, namely, enantiopure crystals.^13^

Hence, it is generally expected that when they are crystallized from racemic mixtures, one would obtain an equal mixture of enantiomorphous crystals of opposite handedness. Resolution of enantiomers by crystallization should confirm that the enantioselective interaction between ferromagnetic substrates and chiral molecules is effective not only upon the formation of chemical bonds^14^ but also for non-covalent interactions. To ensure that the results obtained are not due to statistical fluctuations we conducted two sets of experiments:

(i) Crystallization of the pure enantiomers of the three amino acids on different FM surfaces where the magnets are pointing either North (N) or South (S).

(ii) Crystallization of the racemic mixture on the FM surfaces with two magnets, one pointing N and the other S, located at different sites of the surface.

Fig. 1 shows the experimental setup. Experiments were performed using a ferromagnetic layer (FM) that is exposed to a constant magnetic field of 0.42 T. The FM layer was prepared by evaporation of 10 nm gold on a 120 nm Ni-coated silicon wafer. It is known that a thin layer of a noble metal like gold (up to about 10 nm), deposited on a FM, does not diminish the magnetic or spin transport properties but protects the FM from oxidation.^15^–^17^ This ferromagnetic layer was chosen so that the easy axis is parallel to the surface plane. Hence, without an external magnetic field, no out-of-plane magnetization is measured. The experiments were performed either with one magnet pointing with its North pole up (N) or with the South pole up (S), or with two magnets underneath the silicon wafer, pointing in opposite directions. Either racemic or enantiopure supersaturated solutions were prepared by dissolving the appropriate molecule in warm water at 80 °C. The crystallization process was initiated when the solution was left to cool down to room temperature. The crystallization process was stopped when the first few crystals appeared on the wafer surface; this could take several hours and up to several days.

**Fig. 2:**
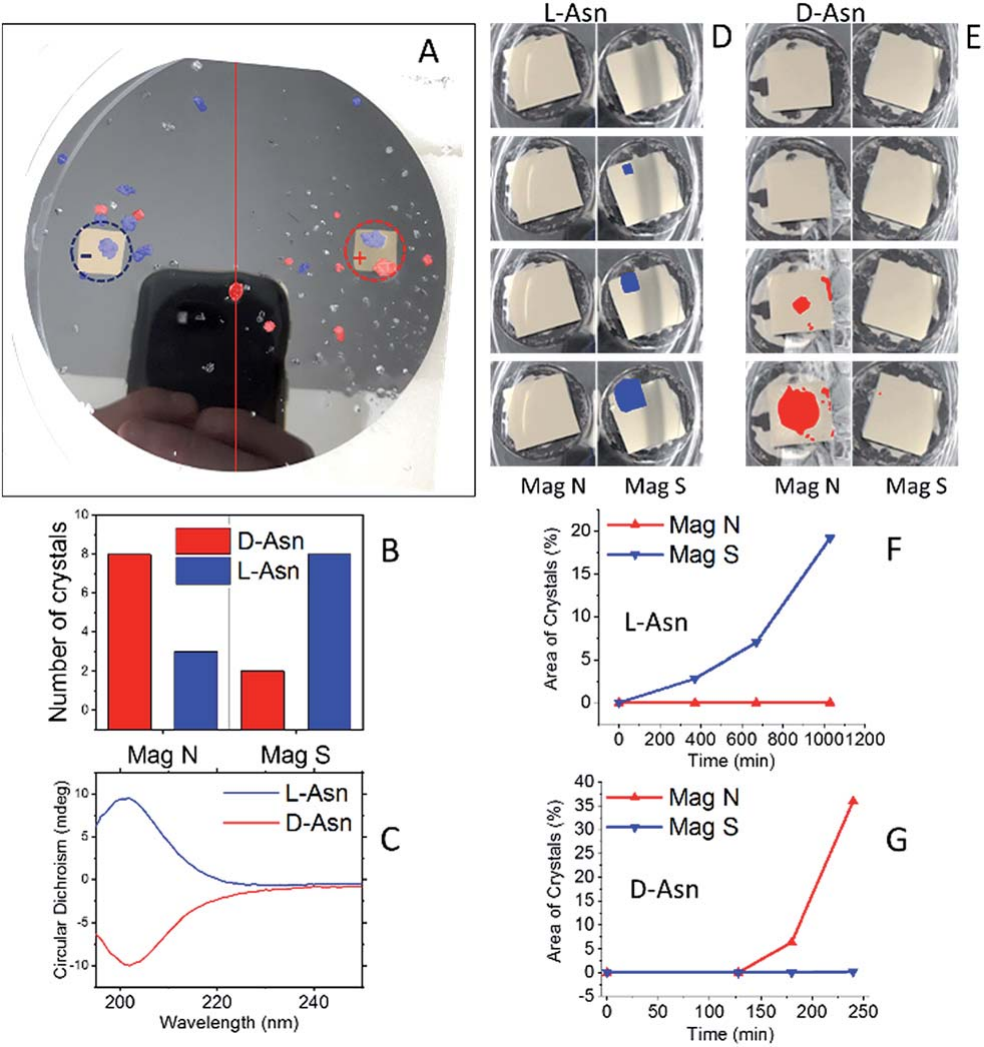
Enantioselective crystallization of asparagine (Asn). (A) A racemic solution of asparagine crystallized on a Ni/Au surface (120 nm/10 nm). In red are d-Asn·H_2_O crystals, in blue are l-Asn·H_2_O crystals, as measured by CD. The dotted lines denote the area of the magnet located underneath. + and – indicate a magnet aligned N or S, respectively. (B) The amount of l-Asn (blue) and d-Asn (red) crystals in each half of the wafer. (C) Typical CD spectra of l-Asn (blue) and d-Asn (red) crystals collected from the substrate. (D) The experiment performed with pure l-Asn using the experimental configuration as shown in Fig. 1C. Crystals are formed first on the magnet pointing S. (E) The experiment performed with pure d-Asn using the experimental configuration as shown in Fig. 1C. Crystals are formed first on the magnet pointing N. The plots (F) and (G) show the growth rate of the crystals on the two magnets, pointing either S or N, for the enantiopure solution of l- or d-Asn, respectively.

Fig. 2 presents the results obtained for Asn. Two experimental configurations were applied, as shown in Fig. 1B and C. In the first one, two magnets were positioned, one pointing N and the other S, in a supersaturated racemic mixture of dl-Asn. The Asn·H_2_O crystals, formed at the end of the crystallization process, were singularly hand-picked, redissolved and analyzed by circular dichroism spectroscopy (CD) and are presented in Fig. 2A, using false colors. The crystals found as having excess of the l enantiomer are presented in blue and those that have an excess of the d enantiomer are presented in red. The enantioselectivity in crystallization is not observed only on the region of the wafer directly above the magnets themselves (where the field is stronger)—actually there is a majority of one enantiomer in the entire half wafer closer to the relevant magnet, as indicated. This is attributed to the formation of small enantiopure crystal seeds, initiated on the magnetized surface, which diffuse away and keep growing until they fall down. Clearly, in such a case one expects to observe enantiomorphous crystals in the proximity of the magnet and not only on the magnet itself.

**Fig. 3:**
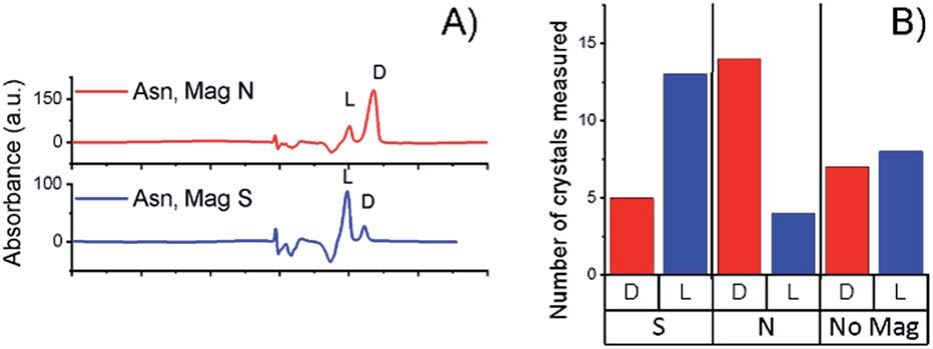
(A) Chiral-HPLC data from a collection of crystals that grew directly on top of the magnets, as shown in Fig. 2A. (B) Histogram showing the handedness of the Asn crystals grown from a racemic solution on top of a surface magnetized either S, N or not magnetized.

Fig. 2B presents the statistics of crystals measured by CD spectroscopy, for each half of the wafer. The CD spectra (an example is shown in Fig. 2C) were taken by dissolving each crystal individually in water; the CD intensity was normalized by the UV-vis absorption peak, so as to correct for differences in concentration. About ten different crystals were collected from each side of the wafer. Clearly, l-Asn tends to crystallize on the S pole of the magnet, whereas d-Asn crystallizes faster on the N pole. The kinetics of the crystallization was monitored when the magnets were positioned in enantiopure solutions. The goal of this part of the experiment was to prove that the enantiospecific crystallization, observed with the racemic mixture, does not result from statistical fluctuations. Four separate sets of experiments were performed (see ESI†) and results were consistent with the experiments performed with the racemic mixture. Indeed, also in this configuration l-Asn crystallized faster on the S pole and d-Asn faster on the N pole. Fig. 2F and G show the area covered with crystals as a function of time, when the crystallization takes place from the enantiopure solutions of l-Asn and d-Asn, respectively. The preferred magnetic pole for each enantiomer is clearly visible. The solutions prepared from the crystals collected directly from the top of the magnets (in Fig. 2A) were also analyzed using chiral HPLC (equipped with an Astec CHIROBIOTIC T, 25 cm × 4.6 mm, 5 μm particle column). The chromatograms are shown in Fig. 3A and indicate an enantiomeric excess (ee) of around 60%, namely, a purity greater than 1 : 4. This result is consistent with the CD intensity, as shown in Fig. 2C.

Crystallization was also performed with a racemic mixture using only a single magnet, to verify that indeed the crystallization is faster in one of the magnet directions, thus demonstrating that the effect observed does not result from statistical fluctuations. The experiment with the dl-Asn and a single ferromagnetic layer in solution was repeated 18 times for each magnet direction, using different batches of substrates and different batches of dl-Asn, and 14 times for the non-magnetized substrates. The unaggregated data and a statistical analysis of the results are presented in the ESI.† Histograms showing the handedness of the asparagine monohydrate crystals that grew on top of a surface magnetized either S, N, or not magnetized (mainly parallel magnetization) are presented in Fig. 3B. The results indicate beyond any doubt that l-Asn·H_2_O crystallized faster on the magnet pointing S, whereas d-Asn·H_2_O crystallized faster when the magnet was pointing N. No preference was measured using the non-magnetized substrate.

**Fig. 4:**
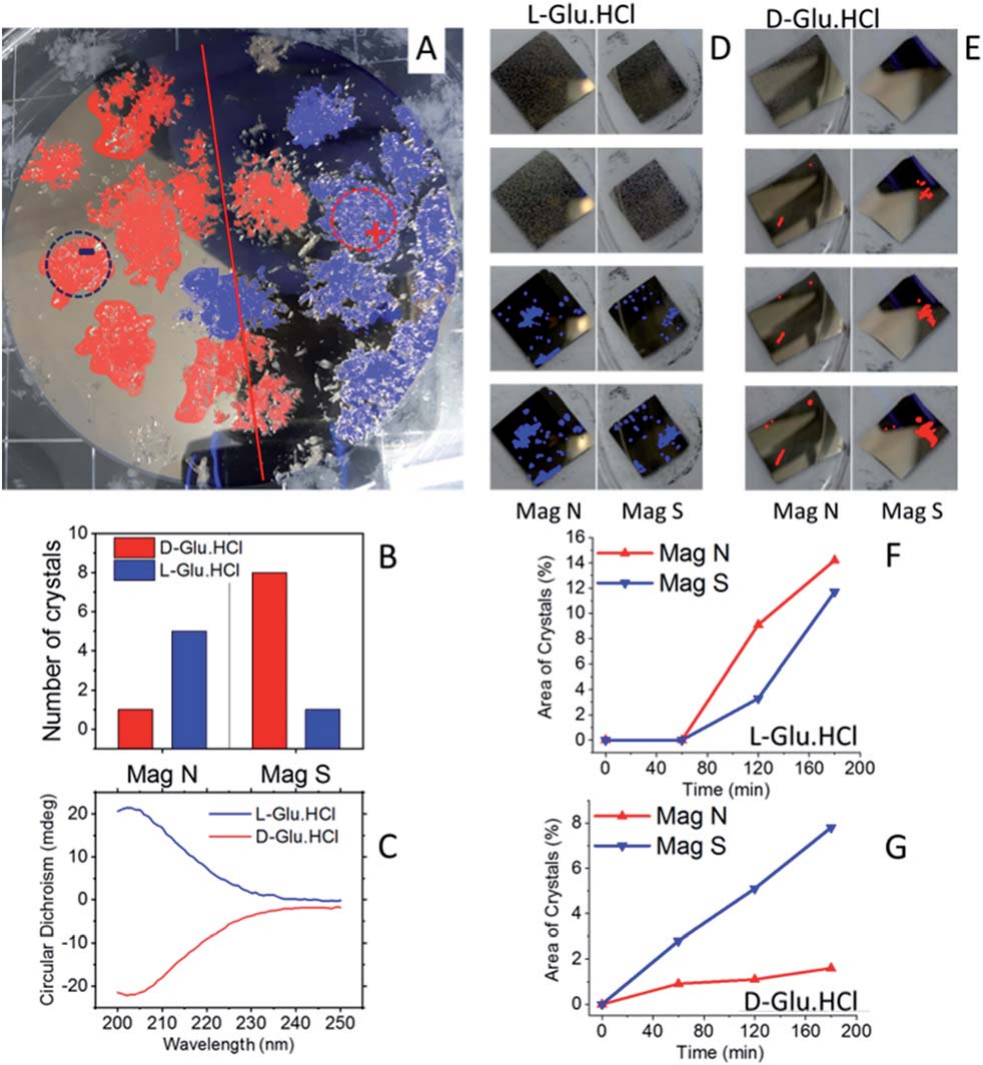
Enantioselective crystallization of glutamic acid hydrochloride. (A) A racemic solution of Glu·HCl crystallized on a Ni/Au surface (120 nm/10 nm). In red are d-Glu·HCl crystals; in blue are l-Glu·HCl crystals, as measured by CD. The dotted lines denote the area of the magnet located underneath. + and – indicate a magnet aligned N or S, respectively. (B) The amount of l- (blue) and d- (red) Glu·HCl crystals in each half of the wafer. (C) Typical CD spectra of l- (blue) and d- (red) Glu·HCl crystals collected from the substrate. (D) An experiment performed with pure l-Glu·HCl using the experimental configuration, as shown in Fig. 1C. Crystals are formed first on the magnet pointing N. (E) An experiment performed with pure d-Glu·HCl using the experimental configuration as shown in Fig. 1C. Crystals are formed first on the magnets pointing S. The plots (F) and (G) show the growth rate of the crystals on the two magnets, pointing either S or N, for the enantiopure solution of l- or d-Glu·HCl, respectively.

The same experiments, as shown in Fig. 2, were conducted with Glu·HCl (see Fig. 4). Results are shown for crystallization from a racemic mixture (Fig. 4A) and from enantiopure solutions (Fig. 4D–E). Clearly, here the d-Glu·HCl crystallized near the S pole, whereas the l-Glu·HCl crystallized near the N pole. The crystallization from enantiopure solutions was performed for glutamic acid in the experimental configuration shown in Fig. 1C. These measurements show that the crystallization was faster for l-Glu·HCl using a magnet pointing N and for d-Glu·HCl using a magnet pointing S.

**Fig. 5:**
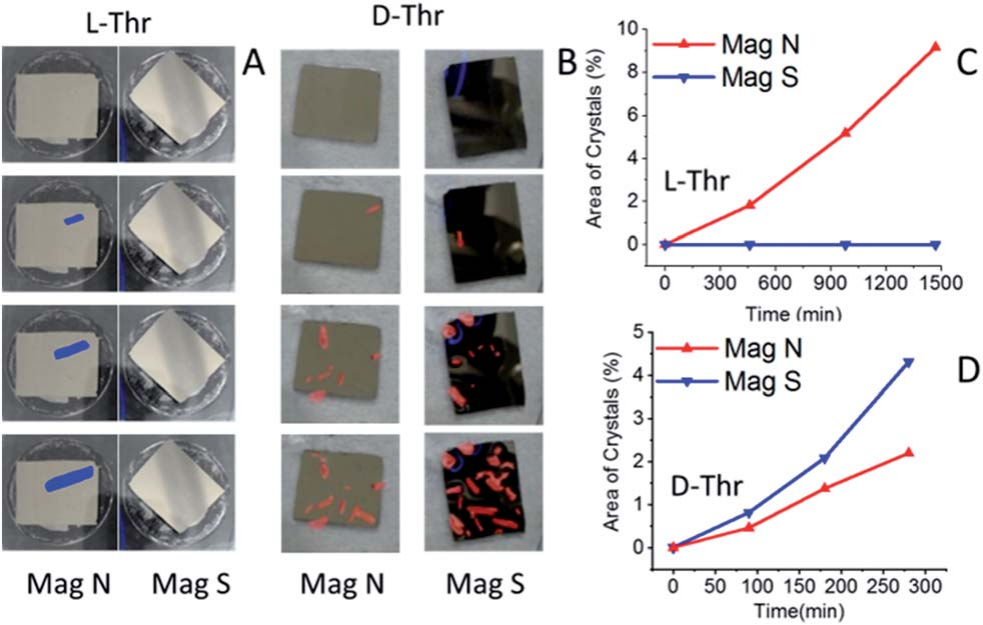
Crystallization of l- and d-Thr from supersaturated enantiopure solutions. (A) Crystallization of l-Thr as a function of time when magnets are pointing N and S. The deposition is faster at the N pole. (B) The same for an enantiopure solution of d-Thr. Here the crystallization is faster at the S pole. (C) The area of magnets covered by crystals as a function of time for a solution of Thr. (D) The same for a solution of Thr.

Despite repeated efforts, we could not obtain Thr conglomerates from a racemic solution. Indeed, it is known that the formation of conglomerate crystals of Thr depends very sensitively on the exact conditions. Apparently it forms twinning crystals of the two enantiomorphs when crystallized from a racemic solution under the conditions of the present study.^18^ However, spin-dependent crystallization was observed when a magnet pointing N or S was placed under the ferromagnetic substrate in an enantiopure solution of Thr. Fig. 5 presents a set of images of the experiment conducted with the FM substrates installed in either l- or d-enantiopure solutions of Thr, as shown in Fig. 1C. Clearly, for l-Thr the crystallization started on the left surface, which is the N pole, whereas for d-Thr the crystallization started on the right magnet, which exposes the S pole.

## Conclusions

The results presented provide support to the notion that chiral molecules interact in an enantiospecific manner with ferromagnetic substrates magnetized perpendicular to their surfaces.^12^,^14^ It is important to emphasize that a magnetic field *per se* cannot cause enantioselective process.^14^ The effect results from a short range spin–spin exchange interaction. Namely, the spin-polarized ferromagnet interacts favorably with enantiomers that have spin polarized in the opposite direction, as shown in Fig. 1D. Consequently, the longer residence time of one enantiomer on the surface, owing to the stronger interaction, increases the probability of crystallization of that enantiomer. Once the crystallization process starts, it is self-catalyzed like any other crystallization process. Based on the current studies, it is impossible to state whether single molecules or rather large clusters, formed in the solution, interact with the FM.^19^,^20^ Further studies attempting to better understand this mechanism might provide additional insight into the mechanism underlying the early stages of crystal nucleation.

The non-ideal separations observed could be caused by several factors. The first is the non-ideal spin polarization in the substrate. It is known that in Ni the ratio between the density of the states of the two spins at the Fermi level is about 1 : 8.^21^ In our study the Ni is coated with 10 nm of gold, to protect Ni from oxidation; this may further reduce the spin polarization. Another parameter that may affect the enantiopurity is the spontaneous crystallization in the solution above the magnetic substrates. The crystals that grew in solution may deposit on the ferromagnetic substrate and may reduce the enantiopurity. However, even as is, the current work provides a new possible approach to utilize the enantioselective crystallization method, which is generic for many molecules that form conglomerate crystals or diastereoisomers.

It is important to note that whereas in the case of Asn and Thr the d enantiomer is crystallized on the North pole and the l enantiomer on the South pole, for Glu·HCl and Thr the selectivity is reversed. In attempting to determine the origin of the difference, we performed DFT calculations (see details in the ESI†) for a cluster formed by a single molecule of asparagine or glutamic acid adsorbed on a gold cluster (28 atoms); the level of the theory is CAM-B3LYP/cc-pVTZ (for C, O, N, H) and LanL2DZ for Au. The results of the geometry optimization procedure (fixed gold coordinates and full optimization of the amino acid coordinates) show that asparagine preferentially interacts with the substrate through the amide moiety (CONH_2_ group). However, glutamic acid interacts favorably through the non-amino acid carboxylic group. Apparently, the difference in the nature of the interaction between these amino acids and the FM substrates affects the spin selectivity of the process.

The present work discloses a new aspect of the enantiospecific interaction between chiral molecules and ferromagnetic surfaces. It introduces the possibility to physically separate enantiopure crystals using this effect. As such it adds a new tool and new insights when one considers enantioselective interaction and separation.^22^,^23^ The present effect suggests that aligned spins, due for example to magnetic surfaces, might provide a mean for mirror symmetry breaking in nature.

## Conflicts of interest

FT, ML, RN, YP filed a patent on magnetic separation of chiral compounds, US application No. 62/636,903.

## Supplementary Material

Supplementary information
