# Supplementary material for: Enantioseparation by crystallization using magnetic substrates
Source: Chem Sci. 2019 Apr 10;10(20):5246–50. doi: 10.1039/c9sc00663j (PMC6540959; doi:10.1039/c9sc00663j)
Supplement: Supplementary file 1 [file SC-010-C9SC00663J-s001.pdf]

Electronic Supplementary Material (ESI) for Chemical Science.
This journal is © The Royal Society of Chemistry 2019

Enantioseparation by crystallization using magnetic substrates

SUPPORTING INFORMATIONS

Francesco Tassinari,[a] Jakob Steidel,[a] Shahar Paltiel,[a] Claudio Fontanesi,[b] Meir Lahav,[c] Yossi Paltiel,[d] Ron Naaman[a]

a) Department of Chemical and Biological Physics, Weizmann Institute, Rehovot 76100, Israel

b) Department of Engineering, DIEF, MO26, University of Modena and Reggio Emilia, 41125 Modena, Italy.

c) Department of Materials and Interfaces, Weizmann Institute, Rehovot 76100, Israel

d) Department of Applied Physics and Center for Nano Science and Nanotechnology, The Hebrew University, Jerusalem 91904, Israel

1. Materials

All amino acids were purchased from Sigma Aldrich and used without further purification. The silicon wafers used as substrates were acquired from Virginia Semiconductor (crystal orientation <100>, thickness 525 ±25 μm, boron doped). The water used in the experiments was purified using a Millipore Synergy® Water Purification System (18.2 MΩ/cm at 25°C). The lab-grade acetone used for the cleaning of the substrates was purchased from Bio-Lab Ltd. (Israel), while the absolute ethanol was purchased from Gadot (Israel). The metals used in the ferromagnetic surface preparation were purchased from Mark Technologies (Israel).

DL-Asparagine crystallization (Fig. 3B of main manuscript)

To ensure the racemic nature of the commercial product, a CD spectrum of the material was taken. The absence of circular dichroism confirmed the racemic nature of the compound. For the crystallization, 10g of DL-Asparagine were dissolved in 100mL of deionized water, warmed up to 80°C and stirred until complete dissolution of the material, forming a supersaturated solution.

The magnetic substrates were prepared by e-beam evaporation on top of a [100] silicon wafer, with a multilayer structure made of 8nm of titanium, 120nm of nickel and 10nm of gold. The surfaces were cleaned by boiling in acetone for 10min and then in ethanol for 10min, and drying them with nitrogen. Afterwards they were put for 15min in a UV-Ox cleaner and then used as substrate for the crystallization. The area of the surfaces was $1cm^2$.

The substrates were placed in a thin plastic petri dish made of polycarbonate, and the petri dish was placed on top of a 0.52T permanent magnet, with the magnetic field normal to the surface of the samples. The field was oriented either up or down relative to the surface depending on the experiment. The magnetic field at the surface of the wafers was measured to be around 0.4T.

The supersaturated DL-asparagine solution was filtered still hot using a 0.22□m pore-size filter of PVDF directly on top of the magnetic substrates. Approximately 2mL of solution were placed in each petri dish, so that the FM surface and all the petri dish surface were completely covered. The solution was left untouched until the first crystal appeared on top of the substrates. The crystal was then removed from the surface using tweezers, quickly

washed with deionized water and then dissolved for the evaluation of the enantiomeric excess.

Important: sometimes the crystals are growing also outside of the FM surface (directly on the petri dish). In those cases, we collected only the crystals on the FM surface. Another common scenario is for the crystals to start growing while floating on the solution. In those cases (at least when we were able to see that the crystals were not growing on the surface), we did not collect the crystals and discarded the experiment. Often, the crystals that we are able to collect are not single crystals, but rather clusters. It has been observed that if the crystals are not single, the enantiomeric excess is usually not very high, which is an indication that both enantiomeric forms of the crystals are present in the cluster, even if one in higher percentage than the other.

In the main manuscript we present an histogram reporting the absolute configuration for the crystals collected from this experiment (Figure3b). Here we show a graph (FigureS1) reporting the circular dichroism of the crystals collected (normalized by their absorbance to take into account the different concentration of the solutions).

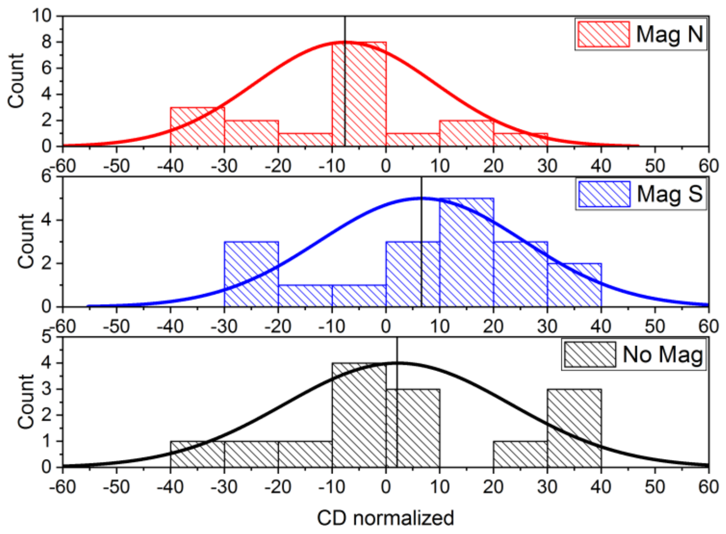

Figure S1: Normalized CD of the crystals collected from the crystallization of DL-Asparagine on a ferromagnetic surface. In red, surface magnetized N; in blue, surface magnetized S; in gray, surface non magnetized.

From the data, we calculate the mean value and the standard deviation for the different magnetization.

Table S1

	Mean CD/Abs	Standard deviation
Magnetization N	-7.6	16.7
Magnetization S	6.6	19.0
No Magnetization	2.1	21.0

If we perform a two sample t-test to see if the two distribution obtained with N and S magnetization have a significantly different mean (null hypothesis, MeanN-MeanS=0) we get that the null hypothesis is rejected with a confidence <0.05.

If we consider that the possible outcome of this experiment is to get a crystal that has either a D- or L-excess, and that in the absence of an external magnetic field the probability of getting either D- or L- should be the same, we can use a binomial distribution to model the probability of getting our results from simple randomness (Figure S2).

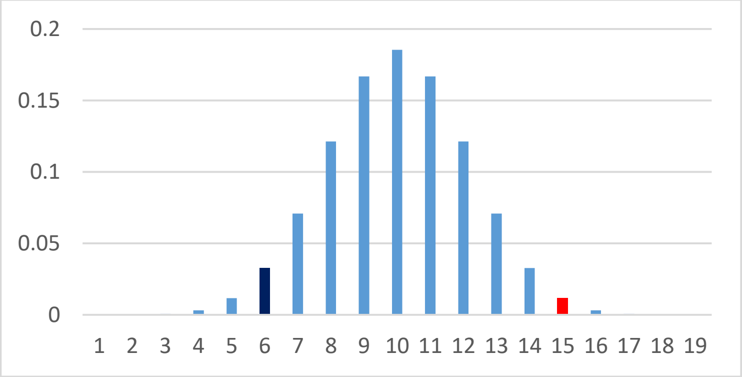

Figure S2: Probability for a binomial distribution (n=18). In red, the probaiblity obtained with magnet pointing N, in blue, the probability for the magnet pointing S.

As it can be seen from the probability histogram, the probability of getting ~78% of D-crystals for Mag N (14 times) and ~72% of L-crystals for Mag S. (13 times) over 18 independent crystallizations gives us a fairly low probability (respectively 1% and 3%) that the results are due to simple randomness in the crystallization process.

DL-Asparagine crystallization (Fig. 2A of main manuscript)

Another similar experiment (Fig. 2A) was done using a large circular substrate having a diameter of 10cm. Two magnets were placed simultaneously near the edges of the wafer, one having the magnetic field pointing up, the other with the field pointing down. The

magnetic layer was evaporated only on the area of the wafer that was going to be placed on top of the magnets. A DL-asparagine solution having a 10g/100mL concentration was filtered hot on top of this surface, as described previously. The system was left untouched until the first few crystals appeared on the surface. They were collected singularly and their position tracked, quickly washed with deionized water and then dissolved for the evaluation of the enantiomeric excess.

In Table S2 we report the CD/Abs value of the various crystals collected and their enantiomeric excess.

Table S2: CD and enantiomeric excess of DL-Asparagine crystals

	CD/Abs (a.u.)	ee (%)	L or D
Crystal 1	+16.1	60.1	L
Crystal 2	+2.2	8.2	L
Crystal 3	+2	7.5	L
Crystal 4	-7.5	-28.0	D
Crystal 5	+6.6	24.6	L
Crystal 6	+7	26.1	L
Crystal 7	-26.8	-100	D
Crystal 8	-24.5	-91.4	D
Crystal 9	+2.7	10.1	L
Crystal 10	-12.3	-45.9	D
Crystal 11	+6.5	24.3	L
Crystal 12	-15.7	-58.6	D
Crystal 13	-10.5	-39.2	D
Crystal 14	-3.3	-12.3	D
Crystal 15	-8.5	-31.7	D
Crystal 16	+6	22.4	L
Crystal 17	+25	93.3	L
Crystal 18	-20	-74.6	D
Crystal 19	-8	-29.9	D
Crystal 20	+26.8	100	L
Crystal 21	+24	89.6	L

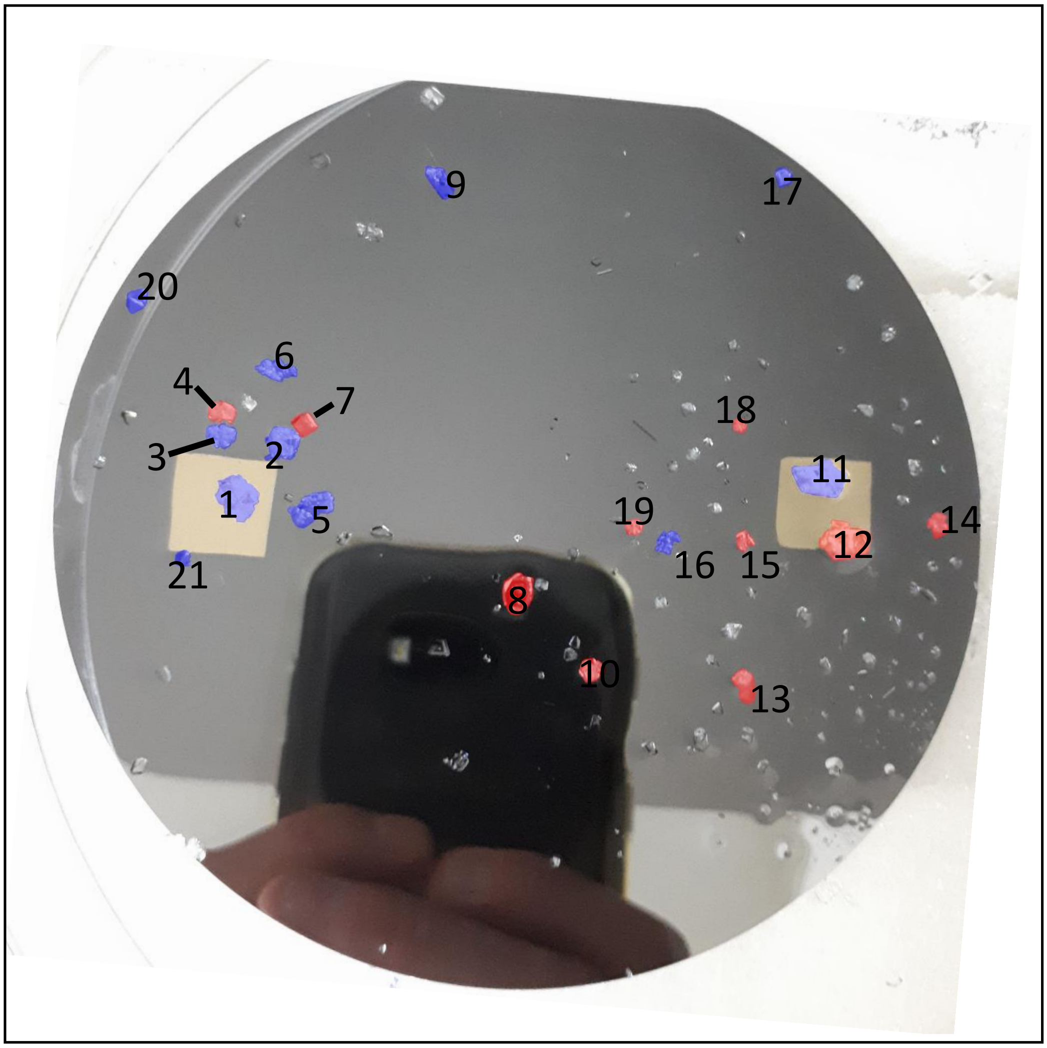

DL-Glutamic acid crystallization (Fig. 4A of main manuscript)

The procedure was the same used for the DL-Asparagine experiment with the large surface, but here the FM layer covers the entire wafer. This was done to reduce the etching of the FM layer, that is sensitive to the very low pH of the solution used. The solution was prepared by dissolving 10g of DL-Glutamic acid in 50mL of a 5M HCl solution.

Table S3:

	CD/Abs	ee (%)	L or D
Crystal 1	-15.1	-5.6	D
Crystal 2	3.8	1.5	L
Crystal 3	-22.4	-8.4	D
Crystal 4	1.8	0.7	L
Crystal 5	-25.7	9.7	D

Crystal 6	1.4	0.5	L
Crystal 7	-8.6	-3.2	D
Crystal 8	19.7	7.4	L
Crystal 9	-55	-20.7	D
Crystal 10	-23.0	-8.6	D
Crystal 11	21.4	8.0	L
Crystal 12	-25	-9.4	D
Crystal 13	-24.4	-9.2	D
Crystal 14	28.4	10.7	L
Crystal 15	35.0	13.2	L

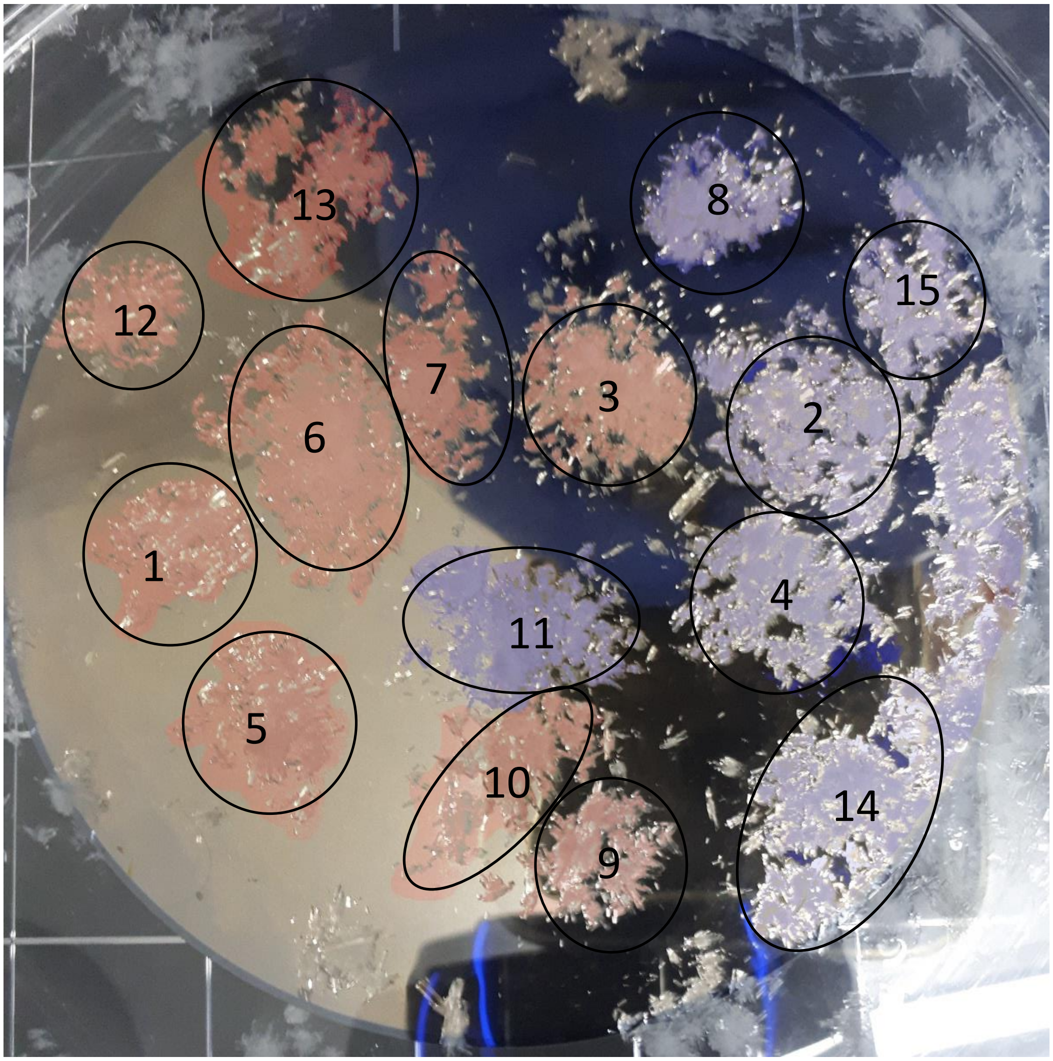

Enantiopure Asparagine crystallization (Fig. 2D-E of main manuscript)

L- and D-Asparagine were crystallized each from a solution of 1.2g in 10mL of deionized water. The crystallization of the enantiopure product was done in a petri dish containing

two magnetic surfaces, each exposed to a 0.42T magnetic field. The two fields were aligned in different directions, one pointing up, the other pointing down. The crystallization dynamics were monitored by taking pictures of the two surfaces at a 5min interval until the end of the crystallization process.

Enantiopure Glutamic Acid crystallization (Fig. 4D-E of main manuscript)

L- and D-Glutamic Acid were crystallized each from a solution of 0.5g in 10mL of deionized water. The crystallization of the enantiopure product was done in a petri dish containing two magnetic surfaces, each exposed to a 0.42T magnetic field. The two fields were aligned in different directions, one pointing up, the other pointing down. The crystallization dynamics were monitored by taking pictures of the two surfaces at a 5min interval until the end of the crystallization process.

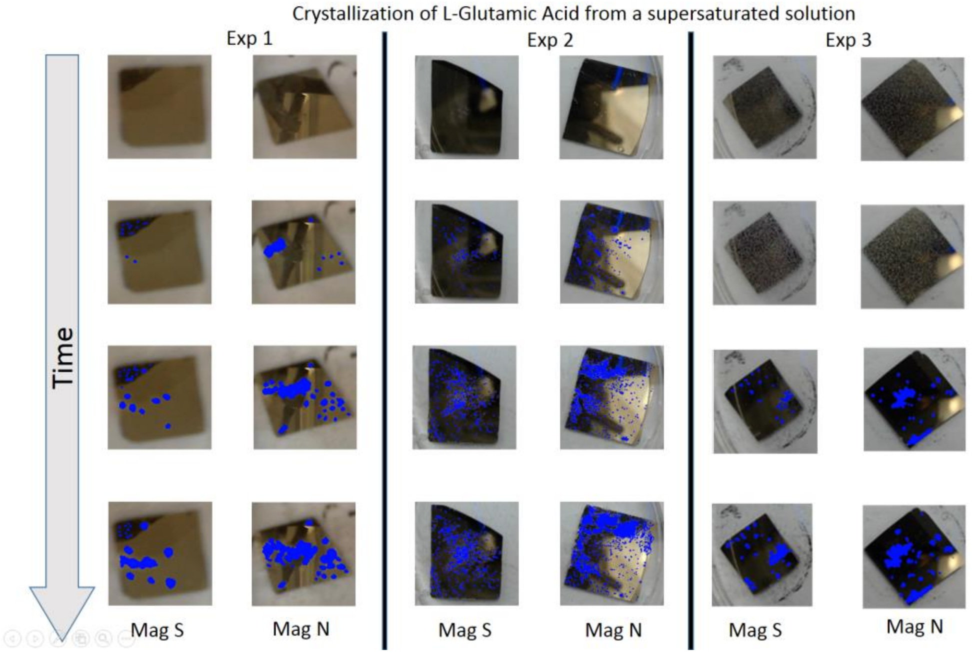

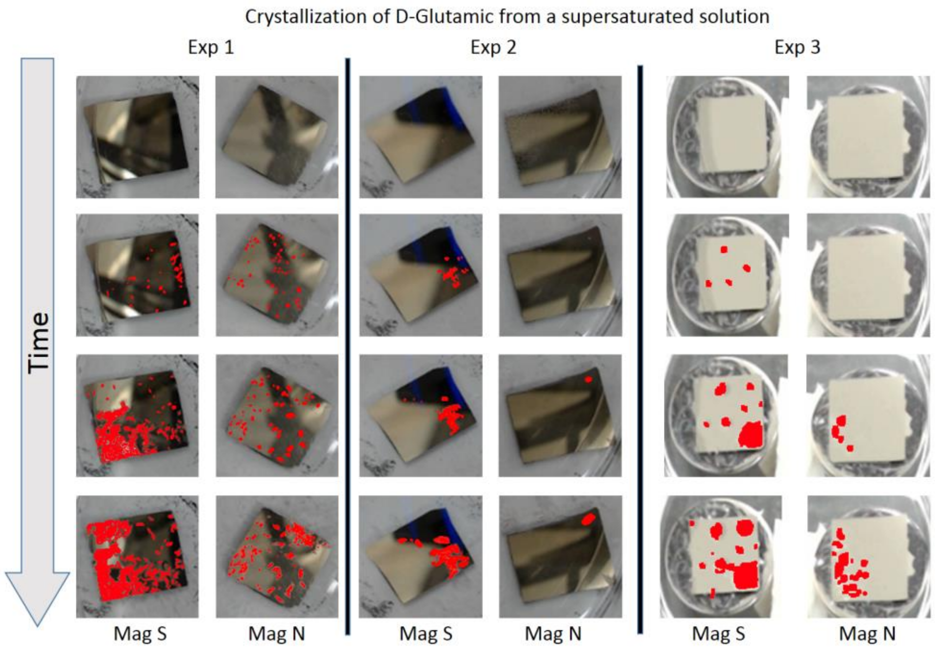

Enantiopure Threonine crystallization (Fig. 5A-B of main manuscript)

L- and D-Threonine were crystallized each from a solution of 1.3g in 10mL of deionized water. The crystallization of the enantiopure product was done in a petri dish containing two magnetic surfaces, each exposed to a 0.42T magnetic field. The two fields were aligned in different directions, one pointing up, the other pointing down. The crystallization dynamics were monitored by taking pictures of the two surfaces at a 5min interval until the end of the crystallization process.

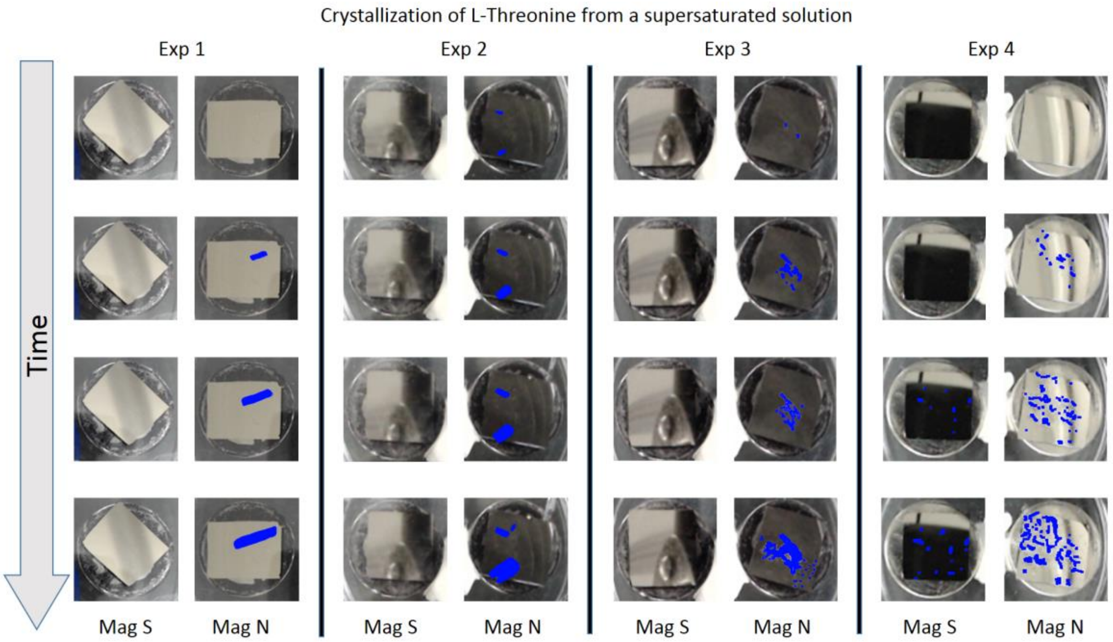

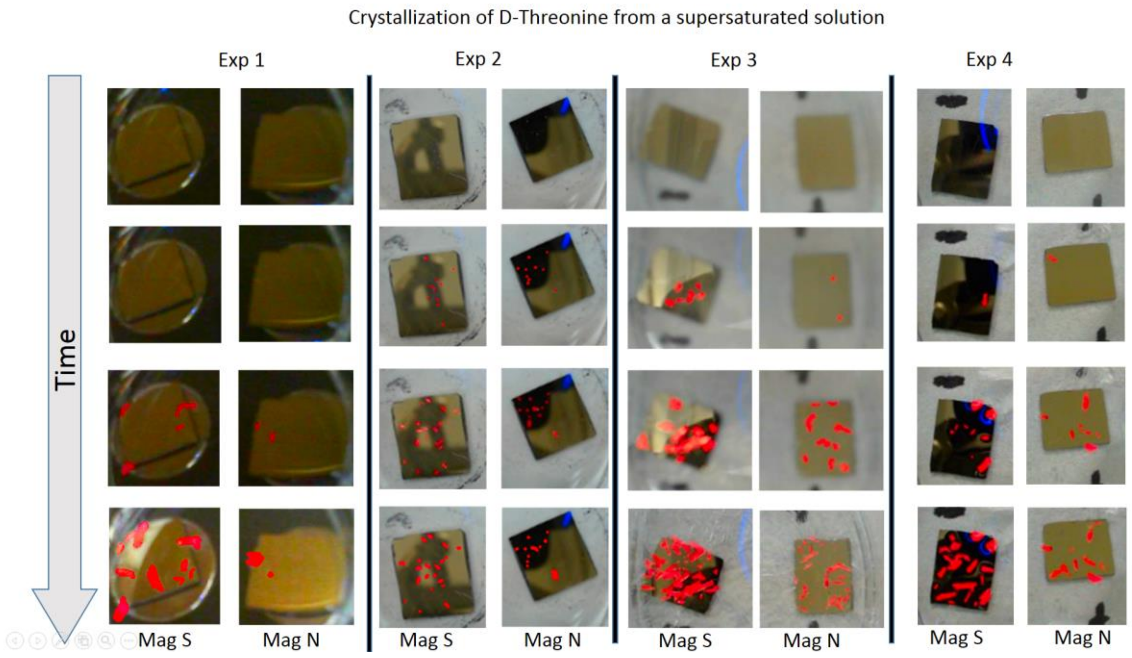

Circular Dichroism spectroscopy

The crystals that grew on top of the surfaces were collected and washed with cold deionized water. They were then dissolved in water and diluted to a concentration of around 1mM. The exact concentration was calculated from the UV-vis absorption spectra, using a calibration curve prepared with L-Asparagine (Fig. S3).

For Glutamic Acid, the procedure was the same but the concentration used was around 30µM. The exact concentration was calculated from the UV-vis absorption spectra, using a calibration curve prepared with L-Glutamic Acid (Fig. S4).

The Circular Dichroism measurements were carried out using a Chirascan spectrometer, Applied Photo Physics, England. The measurement conditions for all spectra were: Scan Range - 200 to 2500 nm; Time per point – 1 second; Step size – 1 nm; Bandwidth – 1nm. The quartz cuvette used had an optical pathway of 1cm.

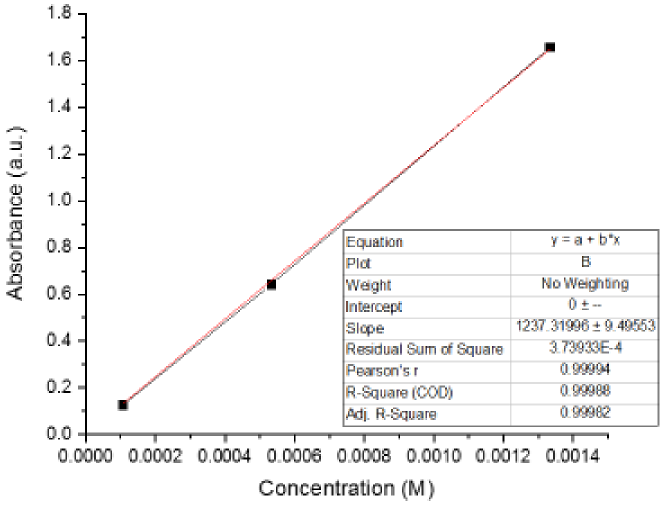

Figure S3: Calibration curve for calculating the concentration of an Asparagine solution from its absorbance.

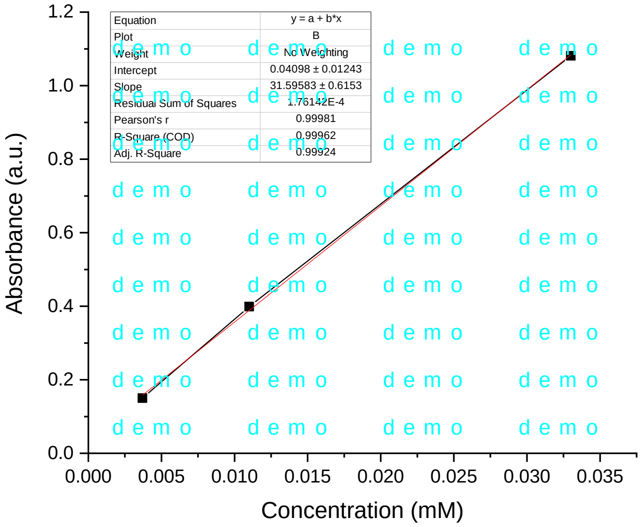

Figure S4: Calibration curve for calculating the concentration of a Glutamic Acid solution from its absorbance.

SQUID

The magnetic properties were measured using a SQUID (superconducting quantum interference device) magnetometer MPMS3 (L.O.T.- Quantum Design inc.) with the magnetic field applied either perpendicular to the sample plane. The vibrating sample

magnetometry (VSM) was used. The measurements were conducted at 300K, and consisted in a magnetizing run from 0Oe to 10kOe, a first measurement going from 10000Oe to -10kOe, a second going from -10kOe to 10kOe, and a demagnetizing run from 10kOe to 0Oe. The diamagnetic contribution of the silicon substrate was measured prior to the Nickel-Gold evaporation and subtracted from the final data.

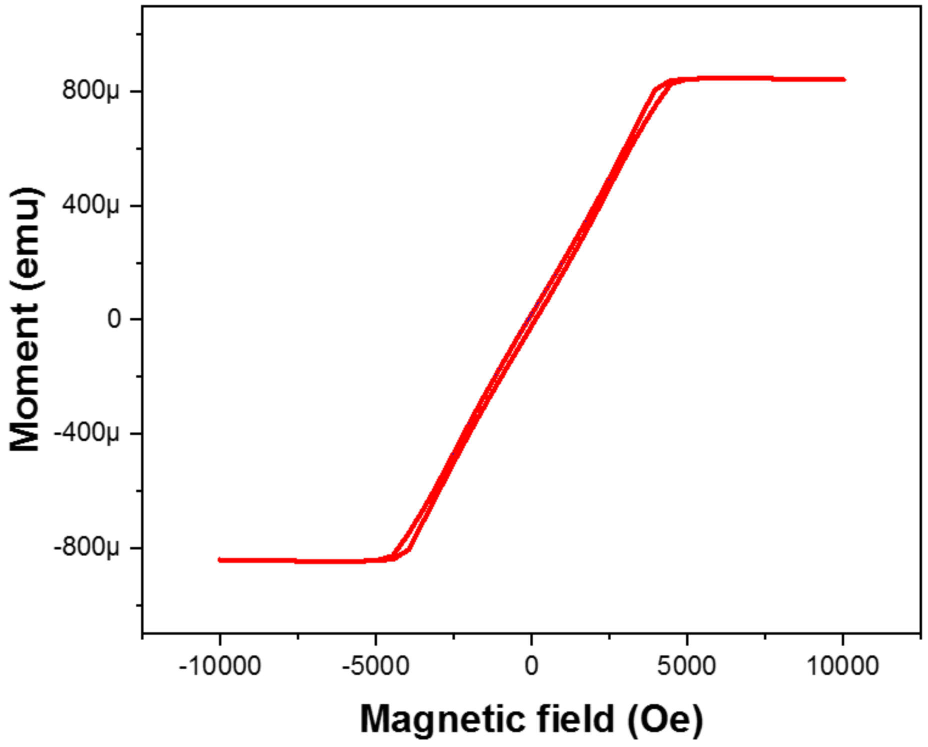

HPLC

The HPLC was an Agilent Technologies 1200 series instrument, equipped with an Astec CHIROBIOTIC T, 25cm x 4.6mm, 5□m particles column. The detector was an UV absorbance detector, used at 205nm wavelength. The mobile phase was a mixture of water/2-propanol 50/50, containing 0.1% of trifluoroacetic acid. The flow rate was 1mL/min. The column was not thermostated.

2. Calculations

Ab initio molecular orbital calculations were performed using the Gaussian 09 and Firefly suite of programs.*(1,2)* Unrestricted CAM-B3LYP density functional theory was used employing 6-31G(d,p) basis sets for the C, H, O, N atoms (a particularly accurate picture is provided by this level of the theory in the analysis of geometrical effects in charged systems *(3*), and Lanl2dz for Au.

The adsorption of L-asparagine (**1**) and L-glutamic acid (**2**) on gold has been modeled using the embedded cluster approach: pursuing the geometry optimization of a single asparagine or glutamic acid molecule in close contact to a gold cluster of finite size, constituted by 22 Au atoms. *(4)*

The gold surface is selected of (111) symmetry, which is the most stable surface orientation for gold.*(5)* To assess the most favorable specific orientation of both asparagine and glutamic acid adsorbed on gold, the energy difference between the two adsorption dispositions was calculated by using DFT based ab-initio calculations: The energy difference between the two adsorption dispositions 1) asparagine pointing towards gold with the NH_2 of the amino group (**1a**), as opposed to the disposition featuring the COOH moiety oriented towards the gold surface (**1b**) 2) glutamic acid pointing towards gold with the carboxylic group near the NH_2 group (**2a**), as opposed to the disposition featuring the COOH moiety, far from the NH_2, oriented towards the gold surface (**2b**). Then, 6-31G(d,p) results have been cross-checked by further optimization using a more accurate basis set for C, H, O, N atoms (cc-pVTZ) and a larger Au (28 atoms) cluster.

The spatial coordinates of the gold cluster were maintained fixed, while the optimization of the asparagine and glutamic acid molecules were carried out without any constrain. Please note that the distance and orientation of both asparagine and glutamic acid, with respect to the gold cluster, were optimized without the application of any further constraint. Due to the extremely large number of conformational degrees of freedom, great attention and a massive endeavor in terms of calculation resources was devoted to a detailed conformational and geometrical screening analysis,where the main problems are the gold cluster dimension and gold cluster/adsorbed molecule reciprocal orientation. Reasonable variations in the equilibrium geometry allow to estimate a sensitivity of about ± 1.5 kcal mol^{-1} for the calculated energy values.

L-asparagine

Figure S5 sets out the L-asparagine/Au(22), amide moiety pointing versus Au, optimized geometry, obtained at the CAM-B3LYP 6-31G(d,p) basis sets for the C, H, O, N atoms and Lanl2dz for Au.

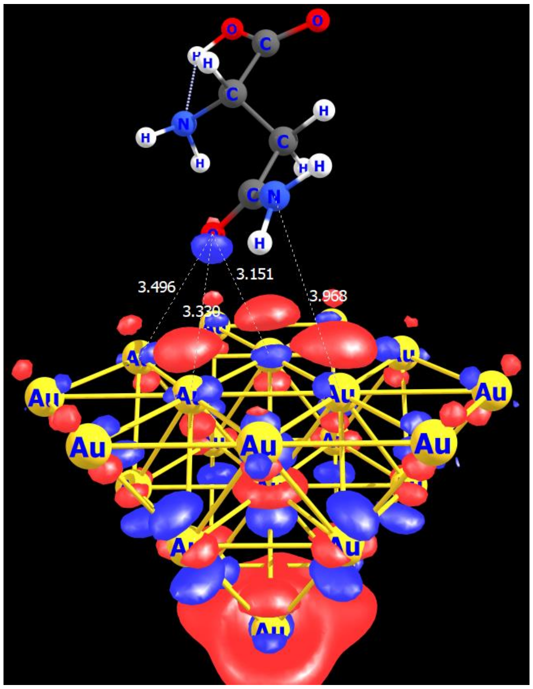

Figure S5: L-Aspargine -NH2, amide moiety pointing versus Au: (**1a**). CAM-B3LYP 6-31G(d,p) basis sets for the C, H, O, N atoms, Lanl2dz for Au. The density distribution of the highest occupied Kohn-Sham orbital is shown

Figure S6 sets out the L-asparagine/Au(22) cluster, carboxylic acid moiety pointing versus Au, optimized geometry, obtained at the CAM-B3LYP 6-31G(d,p) basis sets for the C, H, O, N atoms and Lanl2dz for Au.

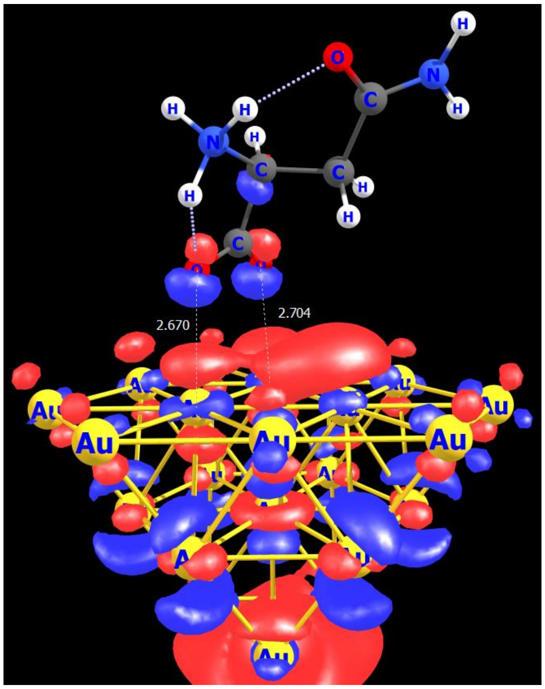

Figure S6: L-Aspargine –COOH moiety pointing versus Au. CAM-B3LYP 6-31G(d,p) basis sets for the C, H, O, N atoms, Lanl2dz for Au. The density distribution of the highest occupied Kohn-Sham orbital is shown.

At the CAM-B3LYP 6-31G(d,p) basis sets for the C, H, O, N atoms, Lanl2dz for Au level of the theory, the adsorption of the L-asparagine featuring the amide group pointing towards the Au cluster, is found more stable by 4.8 kcal mol^{-1}.

These results have been cross checked by further refined in the geometry optimization pursued by using the more accurate cc-pVTZ basis set for the C, H, O, N atoms. The relevant optimized geometries are shown in Figure S6.

(a)

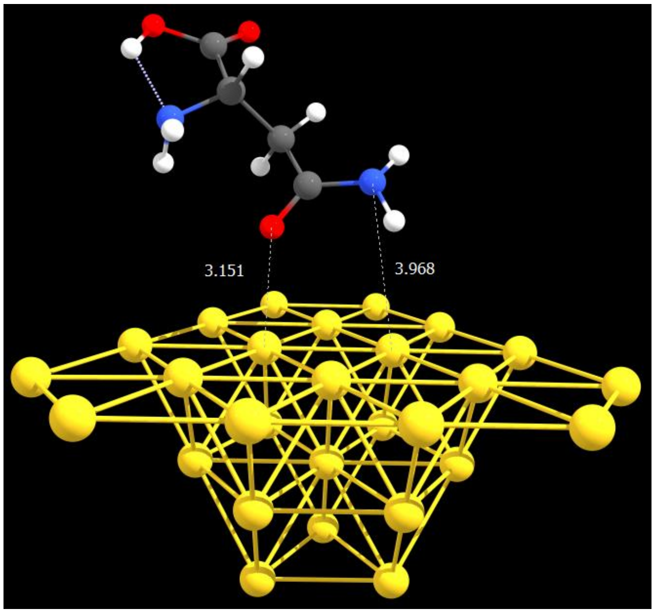

(b)

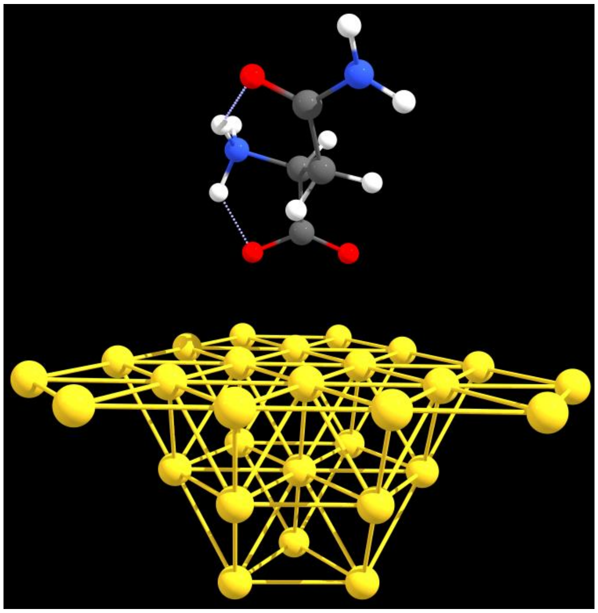

Figure S7: Optimized geometries CAM-B3LYP/cc-pVTZ basis sets for the C, H, O, N atoms, Lanl2dz for Au. a) L-Aspargine -NH2, amide moiety pointing versus Au. b) L-Aspargine –COOH moiety pointing versus Au.

The optimization procedure, starting from guess initial geometries as reported in Figure S5 and S6 confirmed that the cluster featuring the amide moiety pointing towards the Au surface, is the most stable, by 11.8 kcal mol^{-1}, with respect to the cluster. Data reported in Table S4 allow for the relevant energy difference calculations.

Notably, in the case of the L-asparagine/Au cluster, with the amide group pointing towards the gold, the HOMO has a weak bonding character between the oxygen of the amide moiety and the gold. On the contrary, when the COOH moiety is pointing towards the Au surface, the HOMO between the oxygen of the carboxylic group and Au has a weak anti-bonding character. Compare the relevant HOMO pattern on Figure S5 and S6.

L-glutamic acid

Figure S8 sets out the L-glutamic acid/Au(22), COOH group near the -NH2 moiety pointing versus Au (**2a**) cluster. Optimized geometry, obtained at the CAM-B3LYP 6-31G(d,p) basis sets for the C, H, O, N atoms, Lanl2dz for Au level of the theory.

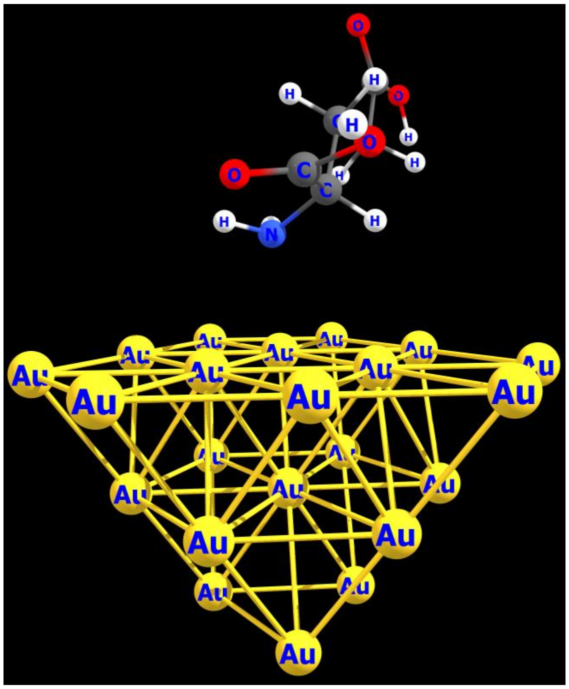

Figure S8: L-glutamic acid. COOH group near the -NH2 moiety pointing versus Au. CAM-B3LYP 6-31G(d,p) basis sets for the C, H, O, N atoms, Lanl2dz for Au.

Figure S9 sets out the L-glutamic acid/Au(22), carboxylic acid moiety far from the NH_2 group, pointing versus Au (**b**), optimized geometry, obtained at the CAM-B3LYP 6-31G(d,p) basis sets for the C, H, O, N atoms, Lanl2dz for Au level of the theory.

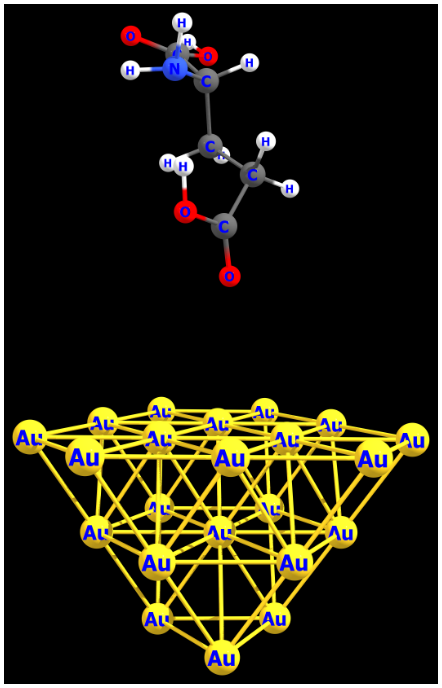

Figure S9: L-glutamic acid. COOH group far from the -NH2 moiety pointing versus Au. CAM-B3LYP 6-31G(d,p) basis sets for the C, H, O, N atoms, Lanl2dz for Au.

At the CAM-B3LYP 6-31G(d,p) basis sets for the C, H, O, N atoms, Lanl2dz for Au level of the theory, the adsorption of the L-glutamic acid featuring the carboxylic group far from the NH_2 group pointing towards the Au cluster, is found more stable by 28.6 kcal mol^{-1}. Again, these results have been cross checked by further refined in the geometry optimization pursued by using the more accurate cc-pVTZ basis set for the C, H, O, N atoms. The relevant optimized geometries are shown in Figure S10.

(a)

(b)

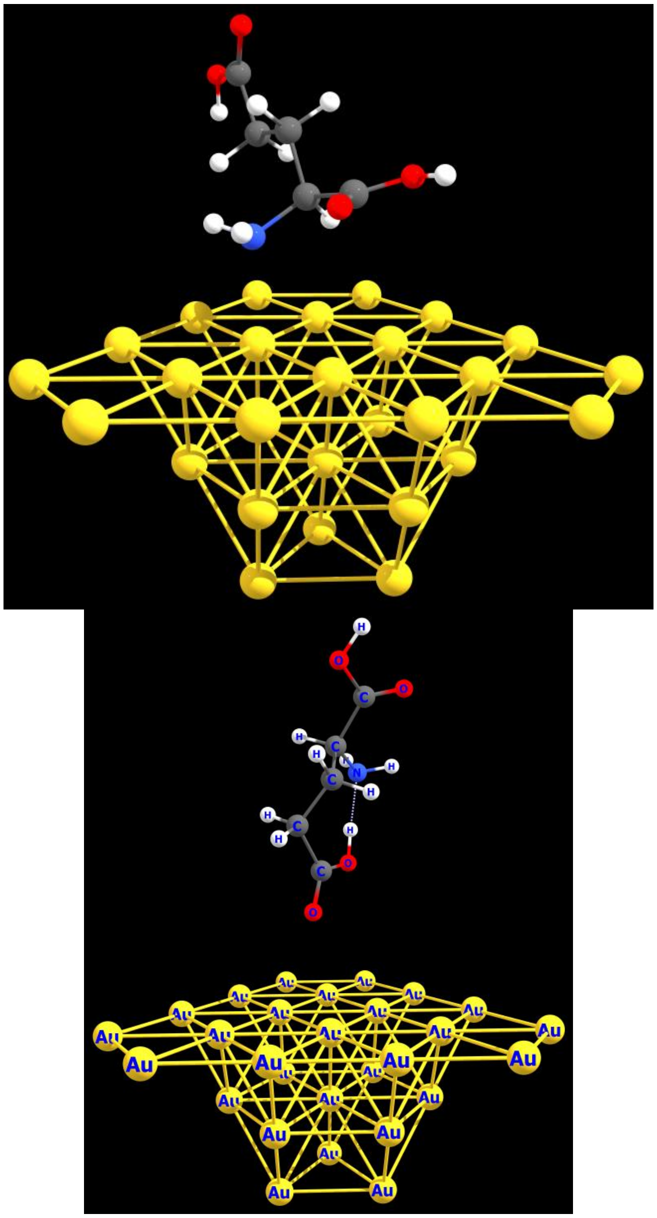

Figure S10: Optimized geometries CAM-B3LYP/cc-pVTZ basis sets for the C, H, O, N atoms, Lanl2dz for Au. a) L-glutamic acid, carboxylic group near the -NH_2 moiety pointing versus Au. b) L-glutamic acid, carboxylic group far rom the -NH_2 moiety pointing versus Au.

The optimization procedure, starting from guess initial geometries as reported in Figure S8 and S8 confirmed that the cluster featuring the carboxylic group far from the NH_2 moiety pointing towards the Au surface (**S10b**), is the most stable, by 8.05 kcal mol^{-1}, with respect to the (**S10a**) cluster. Data reported in Table S4 present the relevant energy difference calculations.

**Table S4.** CAM-B3LYP theoretical energies for the amino acid-gold cluster obtained using two different quality basis sets.	
	Energy
	a.u,
L-asparagine	
6–31G(d,p)	
(1a): –CO–NH_2/Au(22)	–3471.30241740
(1b): –COOH/Au(22)	–3471.29469801
cc-pVTZ	
(1a): –CO–NH_2/Au(22)	–4283.96462723
(1b): –COOH/Au(22)	–4283.94590433
L-glutamic acid	
6–31G(d,p)	
(2a): -(NH_2)COOH/Au(22)	-3530.40311859
(2b): –COOH/Au(22)	-3530.44877076
cc-pVTZ	
(2a): – (NH_2)COOH/Au(28)	–4343.12912476
(2b): –COOH/Au(28)	–4343.14193104

References

(1) Pople, J.A. Gaussian suite of programs, Wallingford, Connecticut, 2017. http://gaussian.com/ (accessed April 18, 2017).

(2) Granovsky A., Firefly version 8.0.0, http://classic.chem.msu.su/gran/firefly/index.html, 2016. http://classic.chem.msu.su/gran/gamess/index.html.

(3) Bruno, C.; Paolucci, F.; Marcaccio, M.; Benassi, R.; Fontanesi, C.; Mucci, A.; Parenti, F.; Preti, L.; Schenetti, L.; Vanossi, D. Experimental and Theoretical Study of the p- and n-Doped States of Alkylsulfanyl Octithiophenes. *J. Phys. Chem. B.* **114,** 8585–8592 (2010). doi:10.1021/jp9122612.

(4) López-Lozano, X.; Pérez, L.A.; Garzón, I.L. Enantiospecific Adsorption of Chiral Molecules on Chiral Gold Clusters. *Phys. Rev. Lett.* **97**, 233401(2006). doi:10.1103/PhysRevLett.97.233401.

(5) Tran, R.; Xu, Z.; Radhakrishnan, B.; Winston, D.; Sun, W.; Persson, K.A.; Ong, S.P. Surface energies of elemental crystals *Sci. Data.* **3,** 160080 (2016).
